# Development of lacosamide for the treatment of partial-onset seizures

**DOI:** 10.1111/nyas.12213

**Published:** 2013-07-16

**Authors:** Pamela Doty, David Hebert, Francois-Xavier Mathy, William Byrnes, James Zackheim, Kelly Simontacchi

**Affiliations:** 1 UCB PharmaRaleigh, North Carolina; 2 UCB PharmaBrussels, Belgium; 3 UCB PharmaSmyrna, Georgia

**Keywords:** lacosamide, partial-onset seizures, epilepsy, antiepileptic drug

## Abstract

Lacosamide is an antiepileptic drug (AED) available in multiple formulations that was first approved in 2008 as adjunctive therapy for partial-onset seizures (POS) in adults. Unlike traditional sodium channel blockers affecting fast inactivation, lacosamide selectively enhances sodium channel slow inactivation. This mechanism of action results in stabilization of hyperexcitable neuronal membranes, inhibition of neuronal firing, and reduction in long-term channel availability without affecting physiological function. Lacosamide has a well-characterized and favorable pharmacokinetic profile, including a fast absorption rate, minimal or no interaction with cytochrome P-450 izoenzymes, and a low potential for drug–drug interactions. Lacosamide clinical development included three placebo-controlled, double-blind, randomized trials conducted in more than 1300 patients, each demonstrating safety and efficacy of lacosamide compared to placebo as adjunctive therapy for adults with POS. The clinical use of lacosamide may broaden, pending results of trials evaluating its use as monotherapy for POS in adults, as treatment for epilepsy in pediatric subjects, and as adjunctive treatment for uncontrolled primary generalized tonic–clonic seizures in those with idiopathic generalized epilepsy.

## Introduction

Epilepsy is the third most common neurological disorder in the United States, with approximately 200,000 new cases diagnosed each year.^1^–^4^ The term *epilepsy* refers to a number of different syndromes characterized by spontaneous or unprovoked disturbances in brain activity with varying characteristics, duration, and severity.^4^ Epilepsy syndromes are classified into two main categories: generalized (seizures beginning in both cerebral hemispheres) or partial (seizures initiating in one specific location).^4^ Though much progress has been made in the diagnosis and characterization of specific seizure types within these broad categories (e.g., simple partial seizures, primarily generalized tonic–clonic seizures), the underlying cellular and molecular mechanisms resulting in seizure activity are largely unknown.^5^

Treatment guidelines for newly diagnosed epilepsy vary, so there is not a standardized approach to epilepsy treatment.^6^–^8^ An area of agreement for most physicians, however, is that the first pharmacotherapy in newly diagnosed patients should be a single antiepileptic drug (AED). When patients fail to achieve seizure control with AED monotherapy, physicians must decide whether additional attempts at monotherapy with other AEDs or introduction of adjunctive AEDs is the best treatment course. While the relative merits of each approach continue to be debated,^9^–^11^ an estimated one-third of patients are unable to achieve adequate seizure control despite the availability of dozens of AEDs.^9^–^11^ Therefore, continued development of treatments for those living with epilepsy is a necessary and worthy endeavor.

## History of lacosamide development

The anticonvulsant properties of lacosamide were characterized by the U.S. National Institute of Neurological Disorders and Stroke (NINDS) Anticonvulsant Screening Program (ASP). On the basis of these screening results, lacosamide was identified as a promising clinical candidate,^12^,^13^ and in 2000, Schwarz Pharma (Monnheim, Germany) and Harris FRC Corporation partnered in the preclinical and clinical development of lacosamide. It was first approved for clinical use in 2008; of note, the availability of multiple lacosamide formulations (oral tablets, syrup, and intravenous (IV) infusion) is distinctive among the AEDs and allows flexibility of administration. UCB Pharma acquired Schwarz Pharma in 2007, and following its approval, lacosamide has been marketed by UCB Pharma or its partners under the brand name VIMPAT®.

The drug discovery process for lacosamide was a combined effort from academia, government agencies, and the pharmaceutical industry. In 1973, Dr. Harold Kohn (an academic chemist now at the University of North Carolina at Chapel Hill) joined the Department of Chemistry at the University of Houston. Dr. Kohn's original scientific interest was in understanding the mechanism of action of biotin, a low molecular weight coenzyme necessary for biological carbon dioxide transfer reactions.^14^ Biotin contains an embedded imidazolidone group, which is a structural unit similar to that found in several AEDs. The potential benefit to drug function through targeted manipulation of chemical structure prompted Kohn to catalog new chemical entities with proven activities in anticonvulsant screens and ultimately led him to propose a novel structural motif (pharmacophore) common in some of these active central nervous system–acting agents. Kohn hypothesized that upon incorporation of this pharmacophore into a new class of compounds, termed functionalized amino acids, a candidate molecule could be synthesized possessing structural specificity along with a favorable anticonvulsant profile. Kohn *et al*. synthesized approximately 250 derivatives of the molecule now known as lacosamide. Lacosamide has a molecular weight of 250.3 Da, and the *R(+)* enantiomer is the active form of the drug.^15^,^16^

## Lacosamide: nonclinical development

Lacosamide demonstrated antiepileptic effectiveness in different rodent seizure models and antinociceptive potential in experimental animal models.^17^ Preclinical electrophysiological studies have demonstrated that lacosamide targets voltage-gated sodium channels and acts by specifically enhancing slow inactivation without affecting fast inactivation of the channel.^18^ Initial preclinical investigations^17^,^19^ suggested that lacosamide might have an additional mode of action by binding to the collapsin response–mediator protein 2 (CRMP-2). However, in a recent publication, Wolff *et al*. demonstrated that there is currently no experimental evidence to support direct binding between lacosamide and CRMP-2.^20^ Lacosamide was also evaluated in a comprehensive preclinical toxicology and pharmacology program^17^ conducted in mice, rats, rabbits, and dogs. These studies found lacosamide to be well tolerated; either no or only minor side effects were observed in safety studies involving the central nervous, respiratory, gastrointestinal, and renal systems, and in three animal models there was no indication of abuse liability. Long-term, repeated-dose toxicity studies demonstrated that after either IV or oral lacosamide administration, adverse events were reversible and consisted mostly of exaggerated pharmacodynamic effects on the central nervous system. No genotoxic or carcinogenic effects were observed *in vivo*, and lacosamide showed a favorable profile in reproductive and developmental animal studies.

## Mechanism of action: a new way to target sodium channels

**Figure 1:**
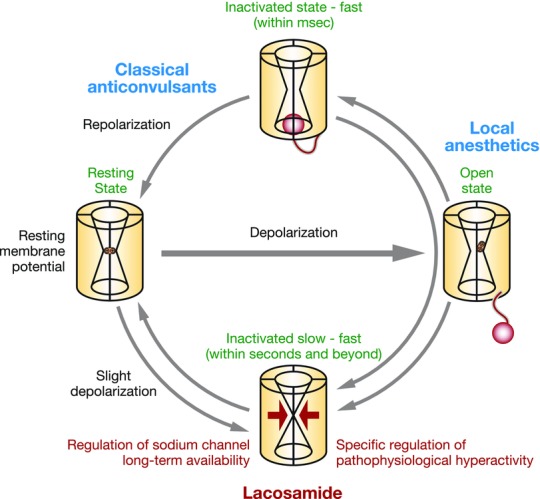
Mechanism of action of lacosamide, classical anticonvulsants, and local anesthetics.

It is well established that voltage-gated sodium channels are responsible for generating action potentials and are therefore intimately involved in controlling neuronal excitability.^21^ In epilepsy, aberrant and repetitive neuronal firing leads to the generation of seizures.^22^ The excitability of the brain depends on the number of sodium channels available for activation.^17^ Two mechanisms regulate the proportion of sodium channels available for activation: fast inactivation (occurring on a millisecond time scale) and slow inactivation (occurring within seconds or minutes). Slow inactivation of voltage-gated sodium channels is induced by sustained membrane depolarization due to repeated neuronal firing (Fig. 1)^17^ like that involved in epilepsy.

Lacosamide was the first AED described to selectively enhance sodium channel slow inactivation, a mechanism of action distinct from traditional sodium channel–blocking AEDs (e.g., carbamazepine, phenytoin, lamotrigine), which predominantly affect fast inactivation (Fig. 1).^18^ Errington *et al*. showed that at clinically relevant concentrations, lacosamide inhibits repetitive fir-ing of neurons characteristic of epilepsy through selective enhancement of sodium channel slow inactivation. This mechanism is thought to be advantageous in that it appears to reduce long-term availability of sodium channels for activation without affecting physiological activity.^17^

## Pharmacokinetic profile

The pharmacokinetic profile of lacosamide includes a fast rate of absorption, dose-proportional plasma concentrations across the approved dose range, minimal cytochrome P450 interaction, and low (<15%) protein binding.^15^,^16^ Maximum lacosamide plasma concentrations occur approximately 1–4 h post-oral dosing, and the elimination half-life is approximately 13 hours. Steady-state concentrations are achieved after 3 days of twice-daily (bid) dosing. Lacosamide is eliminated primarily by renal excretion and biotransformation. Importantly, studies have confirmed that IV lacosamide is bioequivalent to the oral tablet when infused over 30 or 60 minutes.^15^,^16^,^23^ In addition, studies have confirmed the bioequivalence of syrup and tablet formulations of 200 mg/day lacosamide.^24^

Given the low protein binding and minimal cytochrome P450 interactions demonstrated by lacosamide, the risk for drug–drug interaction is low. Lacosamide has no significant effect on plasma levels of other AEDs (e.g., carbamazepine, levetiracetam, lamotrigine, topiramate, valproate, zonisamide, gabapentin, and phenytoin).^15^,^16^,^25^,^26^ In addition to a lack of pharmacokinetic effects on other AEDs, studies have shown no effect on the pharmacokinetics of digoxin, metformin, omeprazole, warfarin, or the oral contraceptive Microgynon® (levonorgestrel/ethinylestradiol) with lacosamide administration.^15^,^16^,^27^,^28^

## Efficacy of lacosamide for partial-onset seizures

Table 1 provides a summary of key lacosamide clinical studies in partial-onset seizures (POS).^29^ The three phase IIb/III registration clinical trials (SP667,^30^ SP754,^31^ and SP755^32^) that served as the basis for regulatory approval of lacosamide shared a similar design (randomized, double-blind, multicenter, placebo-controlled, 12-week studies) and eligibility criteria (Table 2). In each trial, enrolled patients were required to have had an average of ≥ four partial-onset seizures per 28 days with or without secondary generalization, despite a stable regimen of one to three concomitant AEDs (one to two in SP667). Following an 8-week baseline phase, a 4- (SP755) or 6-week (SP667 and SP754) titration period initiated with either placebo or lacosamide 100 mg/day and continued with 100-mg/day weekly increments. Patients randomized to lacosamide 200 mg/day received placebo during the first two (SP755) or four (SP667) weeks of titration. Patients randomized to lacosamide 400 mg/day received placebo for 2 weeks in SP667. One back-titration of 100 mg/day was allowed at the end of titration in cases of intolerable adverse events. The titration phase was followed by a 12-week maintenance phase.

**Table 1:** Summary of key lacosamide clinical trials in POS

Study	Design	Lacosamide treatment	Status
Phase IIb/III efficacy and safety registration studies with oral lacosamide as adjunctive therapy in adults with partial-onset seizures			
SP667^30^	Multicenter, multinational, double-blind, placebo-controlled, randomized, dose–response study	Adjunctive, oral tablet 200, 400, or 600 mg/day	Complete; manuscript published
SP754^31^ (NCT00136019)	Multicenter, randomized, double-blind, placebo-controlled, parallel-group study	Adjunctive, oral tablet 400 or 600 mg/day	Complete; manuscript published
SP755^32^ (NCT00220415)	Multicenter, international, randomized, double-blind, placebo-controlled, parallel-group study	Adjunctive, oral tablet 200 or 400 mg/day	Complete; manuscript published
EP0008 (NCT01710657)	Multicenter, double-blind, randomized, placebo-controlled, parallel-group study in Japanese and Chinese adults	Adjunctive, oral tablet 200 or 400 mg/day	Recruiting
Long-term safety studies with oral lacosamide as adjunctive therapy in adults with partial-onset seizures			
SP615^36^ (NCT00552305)	Open-label, uncontrolled extension (up to 8 years) of SP667	Adjunctive, oral tablet 100–800 mg/day	Complete
SP774^37^ (NCT00515619)	Open-label, uncontrolled extension (up to 5.5 years) of SP755	Adjunctive, oral tablet 100–800 mg/day	Complete
SP756^35^ (NCT00522275)	Open-label, uncontrolled extension (up to 6 years) of SP754	Adjunctive, oral tablet 100–800 mg/day	Complete; manuscript published
SP926 (NCT00655486)	Open-label, uncontrolled extension (up to 2 years) of SP925	Adjunctive, oral tablet 100–800 mg/day	Complete
Phase II/III safety studies of IV lacosamide as replacement for oral lacosamide in adults with partial-onset seizures			
			
SP616^38^ (NCT00800215)	Multicenter, randomized, double-blind, double-dummy, placebo-controlled, inpatient study	Adjunctive, oral tablet or IV infusion (30 or 60 min); daily dose equivalent to current daily dose in open-label extension trial (200–600 mg/day)	Complete; manuscript published
SP757^23^ (NCT00151879)	Multicenter, open-label, serial cohort study	Adjunctive, oral tablet or IV infusion (10, 15, or 30 min); daily dose equivalent to current daily dose in open-label extension trial (200–800 mg/day)	Complete; manuscript published
Safety and tolerability study of adjunctive lacosamide IV loading dose in lacosamide-naive adults with partial-onset seizures			
SP925^39^ (NCT00655551)	Multicenter, open-label, sequential cohort, loading-dose study	Adjunctive, single IV loading dose followed 12 h later with oral tablet for 6.5 days	Complete; manuscript published
Safety and pharmacokinetic studies with oral lacosamide syrup as adjunctive therapy in children with partial-onset seizures			
SP847 (NCT00938431)	Multicenter, open-label study	Adjunctive, oral solution (syrup) up to 12 mg/kg/day	Recruiting
SP848 (NCT00938912)	Multicenter, open-label extension study	Adjunctive, oral solution (syrup) 2–12 mg/kg/day or oral tablets 100–600 mg/day	Recruiting
SP1047	Multicenter, open-label study in subjects 1–17 years of age	Varying doses as prescribed by treating physician	Recruiting
Phase III efficacy and safety studies of oral lacosamide as monotherapy in adults with partial-onset seizures			
SP902/ALEX-MT(NCT00520741)	Multicenter, randomized, double-blind, historical-control study of conversion to lacosamide monotherapy	Conversion to monotherapy, oral tablet 300 or 400 mg/day	Complete
SP0993 (NCT01243177)	Multicenter, randomized, double-blind, double-dummy, positive-control study comparing lacosamide to controlled-release carbamazepine as initial monotherapy in adults with POS or general tonic–clonic seizures	Monotherapy, oral tablet 200–600 mg/day	Recruiting
Long-term safety studies of oral lacosamide as monotherapy in adults with partial-onset seizures			
SP904 (NCT00530855)	Open-label extension (up to 2 years) of SP902	Monotherapy, oral tablet 100–800 mg/day	Active; not recruiting
SP0994 (NCT01465997)	Double-blind extension (up to 3.5 years) of SP0993	Monotherapy, oral tablet 200–600 mg/day	Recruiting

**Table 2:** Design of LCM phase II/III clinical trials

		SP754^31^	SP755^32^
Registration trial	SP667^30^	(NCT00136019)	(NCT00220415)
Phase	IIb	III	III
Total randomized (*n*)	418	405	485
Treatment groups (LCM mg/day)	PBO, 200, 400, 600	PBO, 400, 600	PBO, 200, 400
Randomization scheme	1:1:1:1	1:2:1	1:1:1
Duration (weeks)			
Baseline	8	8	8
Titration	6	6	4
Maintenance	12	12	12
Concomitant AEDs (*n*)	1–2	1–3	1–3
Primary variables	Change in seizure frequency per 28 days from baseline to maintenance phase (presented as median percent reduction), and proportion of patients with at least a 50% reduction in seizure frequency from baseline to maintenance phase (≥50% responder rate)		
Patient-reported outcomes	QOLIE-31^a^	QOLIE-31, PGIC, SSQ	QOLIE-31, PGIC, SSQ

	SP615^36^	SP756^35^	SP774^37^
Long-term extension trials	(NCT00552305)	(NCT00522275)	(NCT00515619)
Dose range	Up to 800 mg/day	Up to 800 mg/day	Up to 800 mg/day
Changes in concomitant AEDs?	Allowed	Allowed	Allowed
New AEDs allowed?^b^	Yes	Yes	Yes

a For SP667, QOLIE-31 was carried out for subjects at U.K. and U.S. sites only.

b New AEDs were allowed to be added only if the subject had not adequately responded to a maximum tolerated dose of LCM.

Note: AED, antiepileptic drug; LCM, lacosamide; PBO, placebo; PGIC, patient global impression of change; QOLIE-31, Quality-of-Life Inventory in Epilepsy; SSQ, seizure severity questionnaire.

All three trials included two primary efficacy variables: change in seizure frequency per 28 days from baseline to maintenance phase (presented as median percent reduction), and proportion of patients with ≥50% reduction in seizure frequency from baseline to maintenance phase (≥50% responder rate). Efficacy was evaluated for the intention-to-treat (ITT) population, which included all randomized patients who received ≥ one dose of trial medication and had ≥ one post-baseline efficacy assessment; also examined was the per protocol set (PPS), which included all randomized patients who received ≥ one dose of trial medication with seizure frequency data from patients in the maintenance phase who had no major protocol deviations. Results from these trials are briefly summarized below.

### SP667 (lacosamide 200, 400, or 600 mg/day versus placebo)

The phase IIb trial conducted by Ben-Menachem *et al*.^30^ included 421 adults with POS randomized 1:1:1:1 to placebo or lacosamide 200, 400, or 600 mg/day. Patients had uncontrolled partial-onset seizures with approximately 84% taking two concomitant AEDs before adding lacosamide to their treatment regimen, and about half of patients having tried ≥ seven AEDs in their lifetime. Despite this difficult-to-treat patient population, treatment with adjunctive lacosamide (400 and 600 mg/day) significantly reduced the seizure frequency compared with placebo as assessed by both primary efficacy variables (ITT and PPS; Table 3). For lacosamide 200 mg/day, statistical significance was achieved for primary efficacy variables in the PPS analysis (Table 3) but not in the ITT analysis (reduction in seizure frequency over placebo was 14.6% (*P* = 0.101); 50% responder rate was 32.7% (*P* = 0.0899)). This was the first published trial evaluating efficacy and safety of adjunctive lacosamide, the results of which supported further development of lacosamide as adjunctive treatment for POS.

**Table 3:** Summary of primary findings from lacosamide phase II/III clinical trials in POS

	ITT population			PPS population		
		
		Seizure			Seizure	
		reduction^a^	50% responder		reduction^a^,^c^	50% responder
	*n*	(median %)	rate^b^ (%)	N	(median %)	rate^b^ (%)
SP667						
Placebo	96	10	21.9		12	21.2
Lacosamide 200 mg/day	107	26	32.7		33*	38.1*
Lacosamide 400 mg/day	107	39**	41.1**		46**	49.4**
Lacosamide 600 mg/day	105	40**	38.1*		49**	49.2**
SP754						
Placebo	104	20.8	18.3		21.7	18.4
Lacosamide 400 mg/day	201	37.3**	38.3**		39.6*	40.0**
Lacosamide 600 mg/day	97	37.8**	41.2**		50.0**	50.9**
SP755						
Placebo	159	20.5	25.8	138	25.4	27.5
Lacosamide 200 mg/day	160	35.3*	35.0	140	35.3*	35.0
Lacosamide 400 mg/day	158	36.4*	40.5**	121	44.9*	46.3**

a *P* values reflect the percent reduction over placebo and are based on log-transformed seizure frequency from pairwise analysis of covariance models with terms for treatment, pooled site, and baseline seizure frequency.

b *P* values are based on a pairwise treatment logistic regression model with terms for treatment and pooled site.

c Data provided for SP667 (UCB Pharma; data on file).

*P* < 0.05; **P ≤ 0.01.

Note: ITT, intent-to-treat; PPS, per protocol set.

### SP754 (lacosamide 400 or 600 mg/day versus placebo)

In this phase III trial conducted by Chung *et al*.,^31^ 405 adults with POS were randomized 1:2:1 to placebo, lacosamide 400 mg/day, or lacosamide 600 mg/day. Most patients (82.1%) were taking two to three concomitant AEDs, and nearly half had tried ≥ seven AEDs in their lifetime. Adjunctive lacosamide (400 and 600 mg/day) significantly reduced the seizure frequency compared with placebo as assessed by both efficacy variables (ITT and PPS; Table 3). A similar reduction in seizures for the primary efficacy analyses was observed for both 400- and 600-mg/day doses with a good balance of efficacy and tolerability for patients randomized to 400 mg/day.

The response to lacosamide was most notable for patients with secondarily generalized tonic–clonic seizures, which constitute the most disabling and potentially harmful seizure type. Specifically, median percent seizure reduction in patients with secondarily generalized seizures at baseline was 93% for 600 mg/day and 59.4% for 400 mg/day compared to 14.3% for placebo. Corresponding responder rates were 70.2% and 56.0% for the lacosamide 600- and 400-mg groups, respectively, compared to 33.3% for placebo.

### SP755 (lacosamide 200 or 400 mg/day versus placebo)

In this phase III trial conducted by Halasz *et al*.*,*^32^ 485 adults with POS were randomized 1:1:1 to placebo, lacosamide 200 mg/day, or lacosamide 400 mg/day. Most patients (86.7%) were taking two to three concomitant AEDs, and over one-third had tried ≥ seven AEDs in their lifetime. Adjunctive lacosamide 200 and 400 mg/day significantly reduced the seizure frequency as evaluated by median percent seizure reduction (Table 3; ITT and PPS). The 50% responder rate for lacosamide 400 mg/day showed statistical significance compared with placebo (Table 3; ITT and PPS) while the 50% responder rate for lacosamide 200 mg/day (35.0% in both the ITT and PPS analyses) was not statistically different from placebo (Table 3; ITT and PPS).

### Pooled analyses of the three phase IIb/III lacosamide trials in POS

#### Pooled population

**Figure 2:**
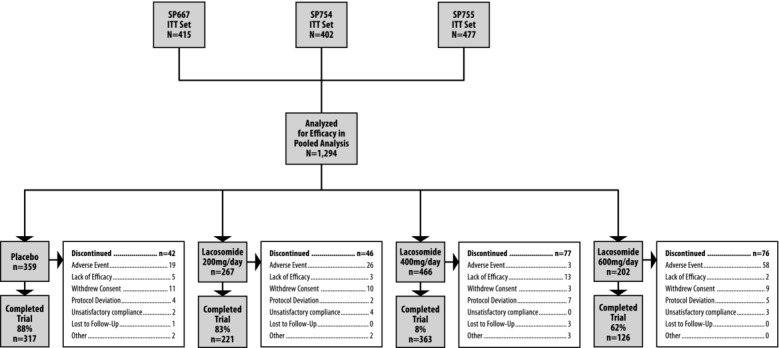
Design of the three lacosamide phase IIb/III studies.

The similar design and patient population of the three lacosamide phase IIb/III studies made them particularly well suited for pooled analyses. In total, 1294 patients received ≥ one dose of trial medication and had ≥ one post-baseline efficacy assessment, comprising the ITT population (Fig. 2). Eighty-eight percent of placebo patients completed the trial compared with 83%, 78%, and 62% of lacosamide 200-, 400-, and 600-mg/day patients, respectively. On average, patients were 38.6 years of age and 51% were female. As mentioned, these patients were a relatively difficult-to-treat group, with use of ≥ four lifetime AEDs in 77% of patients. In addition, the majority (84%) were receiving ≥ two concomitant AEDs, among the most common of which were carbamazepine (35%), lamotrigine (31%), and levetiracetam (29%). The median baseline seizure frequency per 28 days was 11.5.

#### Change in seizure frequency (pooled population)

**Figure 3:**
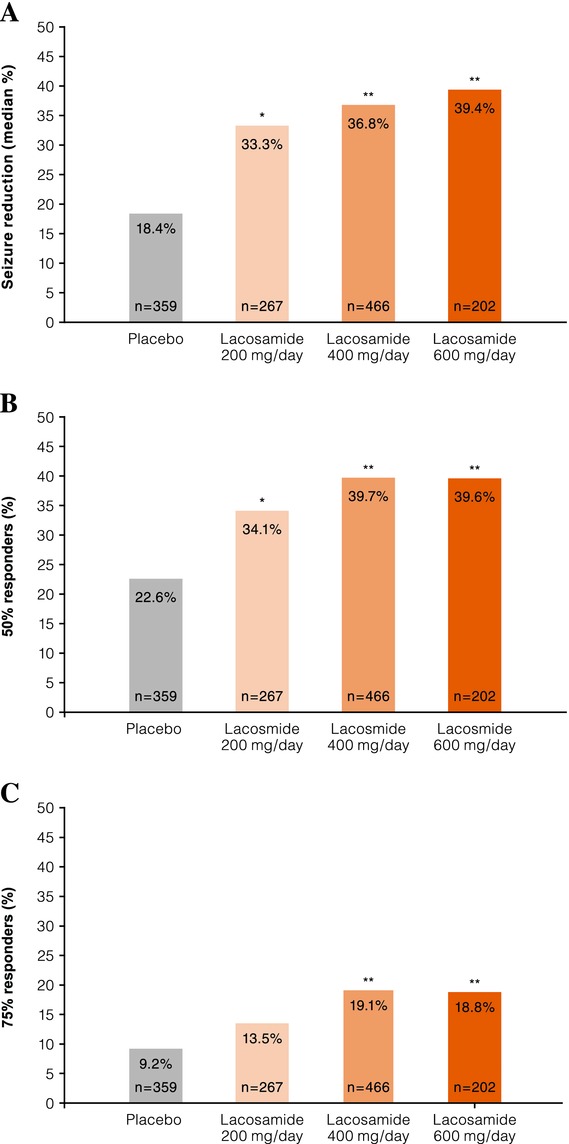
Pooled analyses of lacosamide phase IIb/III clinical trials. Shown is the change in seizure frequency per 28 days (A) and the proportion of patients experiencing a ≥50% (B) or 75% (C) reduction in the seizure frequency following treatment with locosamide or placebo.

Pooled analyses of lacosamide phase IIb/III clinical trials included a priori–defined primary variables for change in seizure frequency per 28 days and the proportion of patients experiencing a ≥50% reduction in seizure frequency (50% responder rate); 75% responder rate was included as an a priori–defined secondary variable. All three lacosamide doses (200, 400, and 600 mg/day) resulted in a statistically significant improvement relative to placebo for both primary variables, and the two highest doses were associated with a statistically significant improvement relative to placebo for 75% responder rates (Fig. 3).

#### Post hoc analyses

Post hoc pooled efficacy analyses were conducted and reported by Chung *et al*.^33^ These analyses included an evaluation of the onset of efficacy and an efficacy assessment based upon surgical history. In the onset analysis, lacosamide efficacy was evident during the first (100 mg/day) and second (200 mg/day) weeks of actual exposure compared to placebo.^33^ When evaluating patients based on a history of prior surgical interventions, lacosamide was found to be equally effective regardless of surgical history.^33^ This is notable as patients with surgical interventions typically reflect a difficult-to-treat population, based upon AED treatment history and baseline seizure frequency of lacosamide phase IIb/III patients with prior surgical interventions for epilepsy.

Sake *et al*. performed a more detailed post hoc analysis of the same pooled patient population^34^ in which patients were grouped based upon the primary mechanism of action of their concomitant AEDs (*n* = 1077 (82%) were receiving ≥ one traditional sodium channel–blocking AED compared with *n* = 231 (18%) who were not). This analysis confirmed previous findings that adjunctive lacosamide resulted in significant seizure reduction relative to placebo, regardless of whether traditional sodium channel blockers were included among the concomitant AEDs.

### Long-term lacosamide studies

Three long-term open-label extensions (SP615, SP756, SP774; Table 2)^35^–^37^ to the phase IIb/III registration studies have been completed. The primary objective was to evaluate safety and tolerability of long-term lacosamide (doses up to 800 mg/day were allowed with exposure for ≤ 8 years). Therefore, the main safety findings from these trials are discussed within the safety section below. Regarding efficacy, median percent reduction from baseline in seizure frequency as well as responder rates were evaluated in each trial and analyzed by completer cohorts. Published results are available for trial SP756,^35^ in which long-term lacosamide (up to 5 years of exposure) was evaluated in patients completing double-blind study SP754 and entering the open-label trial. Median percent reduction in seizure frequency over the yearly interval for 1-, 2-, 3-, and 4-year completer cohorts was 53.4%, 55.2%, 58.1%, and 62.5%, respectively, and efficacy appeared to be sustained over time. Similar results were observed for 50% responder rates in that the percentage of 50% responders across all lacosamide modal doses for 1-, 2-, 3-, and 4-year completer cohorts was sustained over time (52.8%, 56.5%, 58.7%, and 62.5%, respectively).

## Safety and tolerability of lacosamide

Safety and tolerability of lacosamide were evaluated in the three phase IIb/III trials as well as in pooled analyses of these trials, in four long-term extension trials, and in studies evaluating the IV formulation.

### Individual phase IIb/III lacosamide trials in POS

Adverse events and discontinuation data reported from the three individual phase IIb/III trials (SP667, SP754, and SP755)^30^–^32^ were similar, with most commonly reported treatment-emergent adverse events (TEAEs) related to the central nervous and gastrointestinal systems. Trial SP667^30^ included lacosamide 200, 400, and 600 mg/day; in that trial, most commonly reported TEAEs (occurring in ≥10% of patients in any randomized group) during the treatment period (titration plus maintenance phases) were dizziness, headache, nausea, fatigue, ataxia, abnormal vision, vomiting, diplopia, somnolence, and nystagmus. TEAEs leading to discontinuation in ≥5% of patients were dizziness, nausea, ataxia, vomiting, and nystagmus. In SP754,^31^ treatment with lacosamide 400 and 600 mg/day produced similar results with the most common TEAEs identified as dizziness, nausea, diplopia, blurred vision, headache, vomiting, tremor, abnormal coordination, somnolence, and nystagmus. The most common TEAEs leading to discontinuation in this trial were dizziness and abnormal coordination. In trial SP755^32^ (lacosamide 200 or 400 mg/day), three TEAEs (dizziness, headache, and diplopia) occurred in ≥10% of patients in any randomized treatment group. Discontinuations due to TEAEs were relatively low in this trial. Common TEAEs leading to discontinuation included diplopia, vertigo, vomiting, and convulsion.

Top-level results of the pooled analysis of safety data from the phase IIb/III lacosamide trials were reported by Chung *et al*.^33^ Dizziness (31% vs. 8%), headache (13% vs. 9%), nausea (11% vs. 4%), and diplopia (11% vs. 2%) were the four TEAEs that occurred during the treatment period at an incidence of ≥10% in the total lacosamide group (all doses combined; N = 944) and greater than placebo. Aside from headache, incidence of these TEAEs appeared dose related, and all occurred with a lower incidence in the maintenance phase compared with the titration phase. The most common reason for discontinuation was TEAEs; those leading to discontinuation in >5% of patients in any group were dizziness and abnormal coordination (both were observed with lacosamide 600 mg/day).

Three serious TEAEs occurred at an overall rate of ≥1% in any group: dizziness (1.5% for lacosamide 600 mg/day vs. 0% for all other groups), nystagmus (1.0% for lacosamide 600 mg/day vs. 0% in all other groups), and convulsion (1.1% for lacosamide 200 and 400 mg/day, 0% for lacosamide 600 mg/day vs. 0.8% for placebo). No serious safety concerns based on hematology or clinical chemistry values were identified across any of the three trials.

An analysis of patients grouped by the primary mechanism of action of concomitant AEDs (sodium channel–blocking AEDs or nonsodium channel–blocking AEDs) was conducted using this same pooled patient population.^34^ Group assignments were based solely on the presence or absence of one or more of four predefined sodium channel blockers (carbamazepine, lamotrigine, oxcarbazepine, or phenytoin derivatives), and the majority of patients were on multiple concomitant AEDs. These findings showed a lack of dose-dependent discontinuations due to TEAEs when lacosamide was added to a regimen including nonsodium channel blockers. While the authors proposed that this suggests a potential for improved tolerability, it was also noted that the population size of the nonsodium channel–blocking group was too small to draw definitive conclusions.^34^

Others have published small case series and retrospective studies also identifying potential differences in tolerability with lacosamide based on the mechanism of action of concomitant AEDs, though variation in how treatment groups were defined based on the mechanism of action complicates the ability to compare findings.^38^–^41^ Regardless, without properly controlled, prospective studies to directly address the potential for differential outcome with lacosamide in combination with individual AEDs, it is not possible to make definitive comparative statements on treatment combinations with lacosamide.

A meta-analysis of 10 lacosamide randomized controlled trials in various indications was performed by Zaccara *et al*.^42^ In this analysis, the risk of experiencing an adverse event significantly differed between lacosamide and placebo groups for the following adverse events: dizziness at 200 mg/day; dizziness, vertigo, abnormal coordination, abnormal vision, nausea, and vomiting at 400 mg/day; and dizziness, vertigo, ataxia, balance disorder, diplopia, fatigue, nausea, vomiting, and tremor at 600 mg/day. Furthermore, although no formal cognitive testing was performed in the randomized controlled trials, lacosamide was not associated with any adverse event obviously related to cognition in this analysis. The authors also noted that the analyses indicate that the tolerability profile of lacosamide is dose dependent. Applicability of these findings to routine clinical practice is limited by a number of factors, including the heterogeneous populations from the studies used, which evaluated outcome as adjunctive therapy in patients with partial-onset seizures or as monotherapy in patients with neuropathic pain, migraine, fibromyalgia, or knee osteoarthritis.

### Long-term studies

Three long-term, open-label studies that were extensions of the original phase II/III lacosamide studies have been completed (SP615 (exposure up to 8 years),^36^ SP756 (exposure up to 5 years),^35^ and SP774 (exposure up to 5.5 years)).^37^ For all of these trials, the primary objective was an evaluation of safety and tolerability, and in each trial, lacosamide doses up to 800 mg/day and changes in concomitant AEDs were allowed.

In the three open-label trials combined, 1054 patients were treated with lacosamide. Of these, 75%, 53%, and 18% of patients were exposed to open-label lacosamide for >1, >3, and >5 years, respectively, with a median modal dose of lacosamide 400 mg/day. The most common TEAEs were dizziness (37.2%), headache, (18.8%), nasopharyngitis (15.75), and diplopia (15.4%). Most TEAEs were mild or moderate in intensity. An additional long-term study (SP926) was an open-label extension of an IV loading dose trial (SP925). As observed with the other long-term studies, no new safety concerns were identified in SP926, which included treatment with oral lacosamide (100–800 mg/day) for up to 2 years. Overall, safety outcomes with lacosamide observed in long-term extension trials are similar to those reported in the short-term trials and indicate a favorable long-term tolerability profile for lacosamide.

### IV lacosamide

The availability of an IV formulation represents an important advantage of lacosamide relative to many other AEDs. The IV formulation is compatible with a number of other solutions, including saline, dextrose, and lactated Ringer's solutions, and has a demonstrated bioequivalence to the oral formulation with no need for dose adjustment.^23^ The safety and tolerability of IV lacosamide were initially evaluated in two studies (SP616,^43^ SP757^23^). Study SP616 (double-dummy, randomized inpatient trial) evaluated safety, tolerability, and pharmacokinetics of IV lacosamide (200–600 mg/day) administered as 60- or 30-min twice-daily infusions as replacement for oral lacosamide in patients with POS. TEAEs were reported in 16/60 patients and included dizziness, headache, back pain, somnolence, and injection-site pain. All were considered mild or moderate in intensity. The tolerability profile of IV lacosamide in this trial was consistent with that observed with oral lacosamide. Study SP757 evaluated IV lacosamide (200–800 mg/day) at 10-, 15-, and 30-min infusion rates. No increases in frequency or severity of AEs were observed with shorter infusion duration or increased days of exposure, supporting the safety of a 15-min infusion duration for temporary replacement of oral lacosamide.

A recent study, SP925^44^ (open-label, multicenter cohort trial), evaluated safety and tolerability of adjunctive lacosamide IV loading dose in lacosamide-naive patients with POS. An IV lacosamide loading dose (200, 300, or 400 mg) was administered over 15 min, followed 12 h later by initiation of oral dosing consisting of half of the loading dose administered twice daily for 6.5 days. All 100 patients enrolled in the trial completed IV lacosamide infusion, supporting the feasibility of rapid initiation. IV loading doses of 200 and 300 mg lacosamide were better tolerated than the 400-mg dose. Most TEAEs occurred within 4 h of infusion start and are reasonably attributed to the rapidity of infusion or dose. Most commonly reported TEAEs were typical for neuroactive drugs and AEDs in particular. Seven patients discontinued prematurely due to TEAEs. In general, the types of TEAEs leading to discontinuation were similar regardless of the time of onset and were consistent with the most frequently reported TEAEs for the whole trial population. Evaluation of changes in laboratory, electrocardiogram (ECG), and vital sign values following IV loading and oral maintenance lacosamide were consistent with the known oral and intravenous safety profile.

## Approval and clinical use

On the basis of the three randomized, placebo-controlled phase IIb/III trials in adults with POS,^30^–^32^ lacosamide was first approved in 2008 (by both the U.S. Food and Drug Administration (FDA) and the European Medicines Agency (EMA)) as adjunctive therapy in the treatment of POS in adults (aged ≥17 years in the United States and ≥16 years in Europe).^15^,^16^,^30^–^32^ The lacosamide solution for infusion is also approved as an alternative for patients when oral administration is temporarily not feasible.^15^,^16^,^30^–^32^

The recommended starting dose of lacosamide is 50 mg twice/day, which should be increased after 1 week to an initial therapeutic dose of 100 mg twice/day.^15^,^16^ Depending on response and tolerability, the maintenance dose can be further increased by 50 mg twice/day every week to a maximum recommended daily dose of 400 mg (200 mg twice/day).^15^,^16^ Lacosamide may be taken with or without food. In accordance with current clinical practice, if lacosamide is discontinued, it is recommended that this be done gradually (e.g., taper the daily dose by 200 mg/week).^15^,^16^ Dose adjustments are in place for patients with severe renal impairment, end-stage renal disease, or on renal dialysis. In Europe (based on a recent addition to the EMA Summary of Product Characteristics), lacosamide treatment may also be initiated with a single loading dose of 200 mg, followed approximately 12 h later by a 100-mg twice-daily (200 mg/day) maintenance dose regimen.

On the basis of blinded clinical studies, open-label extensions, and postmarketing experience, precautions are in place for use of lacosamide due to the potential for dizziness, prolongation of the PR interval, second-degree or higher heart block, and atrial fibrillation and flutter.^15^,^16^

## Conclusion: place in epilepsy treatment and future directions

The commitment of Dr. Harold Kohn and colleagues along with cooperation among government agencies and the pharmaceutical industry ultimately led to the clinical availability of lacosamide, an AED with a mechanism of action unlike traditional AEDs, for the adjunctive treatment of POS in adults. Lacosamide is associated with an ease of use given its favorable pharmacokinetic profile, lack of drug–drug interactions, and availability in multiple formulations (tablet, syrup, and IV). It has been extensively studied and has established efficacy and safety profiles in the treatment of POS. The effectiveness of lacosamide in POS supports a role for the proposed mechanism of action involving the modulation of slow inactivation of sodium channels as a therapeutic target in epilepsy and could lead to the development of additional treatment options for patients with this condition.

UCB Pharma continues efforts to support the availability of lacosamide to patients worldwide. Also, the clinical utility of lacosamide may broaden depending on the results of additional clinical trials evaluating its safety and efficacy as a monotherapy for POS in adults, adjunctive therapy for epilepsy in pediatric subjects, and as adjunctive treatment for uncontrolled primary generalized tonic–clonic seizures in those with idiopathic generalized epilepsy.
